# Data of SSRs primers for high-throughput genotyping-by-sequencing (SSR-Seq) based on the partial genome assembly of *Eugenia klotzschiana* (Myrtaceae)

**DOI:** 10.1016/j.dib.2023.108917

**Published:** 2023-01-19

**Authors:** Leonardo C.J. Corvalán, Larissa R. Carvalho, Amanda A. Melo-Ximenes, Cíntia P. Targueta, Ramilla S. Braga-Ferreira, Rhewter Nunes, Mariana P.C. Telles

**Affiliations:** a Laboratório de Genética & Biodiversidade, Instituto de Ciências Biológicas, Universidade Federal de Goiás, Goiânia, GO, Brazil; b Instituto de Ciências Exatas e Naturais, Universidade Federal de Rondonópolis, Rondonópolis, MT, Brazil; c Instituto Federal de Goiás - Campus Cidade de Goiás, Goiás, GO, Brazil; d Escola de Ciências Médicas e da Vida, Pontifícia Universidade Católica de Goiás, Goiânia, GO, Brazil

**Keywords:** Cerrado, Nuclear genome, Microsatellite, Molecular markers, Population genomics

## Abstract

The neotropical fruit plant *Eugenia klotzschiana* Berg. is endemic from South America and occurs in the Brazilian savannah areas, a biome threatened by intensive agriculture. This species is a plant listed on the Brazilian list of Plants for the Future. The *E. klotzschiana* fruits have great nutritional value and antioxidant activity and are consumed *in natura* or processed into juice or jelly. However, their harvest is predominantly in native areas and needs further studies for large-scale commercialization. Nuclear genomic data and population genetic tools are still quite scarce for the species. Here, we provide data on the first partially assembled genome of *E. klotzschiana* (211 Mbp, ∼75.16% genome coverage, N50 = 3,407, and 46.8% BUSCO completeness), the raw Illumina sequencing reads, and two sets of primers for microsatellite (SSRs) high-throughput genotyping-by-sequencing (SSR-Seq) identified in the nuclear genome. These genomic resources are fundamental for this species conservation strategies and the development of a future breeding program.

**Specifications Table**

**Table:** 

Subject	Biology
Specific subject area	Genomics, Horticultural science
Type of data	Whole-genome sequence raw data, partial genome assembly and primers designed and evaluated in silico for candidate microsatellites markers for high-throughput genotyping-by-sequencing (SSR-Seq)
How the data were acquired	High-throughput DNA sequencing (Illumina MiSeq).
Data format	Raw sequence reads (fastq), partial genome assembly (fasta), and primers (table in the paper).
Description of data collection	Leaves were collected from a specimen of E. klotzschiana (voucher ID: UFG0020120 - Herbarium UFG. and DNA was extracted through leaf tissue with CTAB 2% protocol. Sequencing was obtained using Illumina MiSeq equipment and the partial genome assembly was determined using SPAdes genome assembler software. SSR regions were identified by both MISA and QDD pipelines. Primers were designed with the QDD pipeline.
Data source location	Senador Canedo, Goiás, Brazil (16°37′32,197″ S, 49°4′22,696″ W)
Data accessibility	The partial genome assembly of Eugenia klotzschiana is available on the NCBI Genome database under the accession number JANUWG000000000 (https://www.ncbi.nlm.nih.gov/assembly/GCA_025175695.1/). The sequence reads we used for assembly are available in NCBI SRA database under the accession number SRR21159655 (https://www.ncbi.nlm.nih.gov/sra/?term=SRR21159655). SSR primer information is on Tables 2 and 3 of this paper.

## Value of the Data

• This dataset provides the first partial genome sequence for *Eugenia klotzschiana* Berg. (Myrtaceae, Myrteae). The *E. klotzschiana* partially assembled genome can be used as an initial reference in several future studies in the areas of evolutionary biology, comparative genomics, and plant breeding. It can also be used on the development of molecular markers for genotyping on a large scale mainly in population genetics and genomics studies;
• The genomic data made available by us is a partial genome assembly for *Eugenia* (∼75.16% % of *Eugenia* mean genome size). Our assembly is the most contiguous and complete genome (considering the number of genes) obtained so far for the genus;
• The primers designed for SSR high-throughput genotyping-by-sequencing are genetic tools that were validated *in silico* and have the potential to be useful in many population genetics applications, after proper laboratory testing, such as genetic characterization of entire populations with less sequencing effort on Illumina Platform. Therefore, it enables fast and concise analysis of genetic diversity for *E. klotzschiana*.

## 1 Data Description

The neotropical species *Eugenia klotzschiana* Berg. (Myrtaceae, Myrteae), popularly known as "pêra-do-cerrado", is an important genetic resource from the Brazilian Cerrado. This species is on the Brazilian list of Plants for the Future [1], as a priority for cultivation and marketing because of its nutrition and antioxidant values. The fruits of *E. klotzschiana* are used in the production of jellies and juices, presenting high economic value. Although *E. klotzschiana* has no record of threatened status in the IUCN, most of its natural occurrence has recently been lost by extensive agriculture. We generated 16,761,276 raw sequencing reads which were used to assemble the first partial genome of *Eugenia klotzschiana* on an Illumina MiSeq equipment. The sequences obtained from the genome assembly were used for the identification and development of a set of primers to access microsatellite loci to be validated in silico and used for genotyping-by-sequencing (SSR-Seq).

**Fig. 1:**
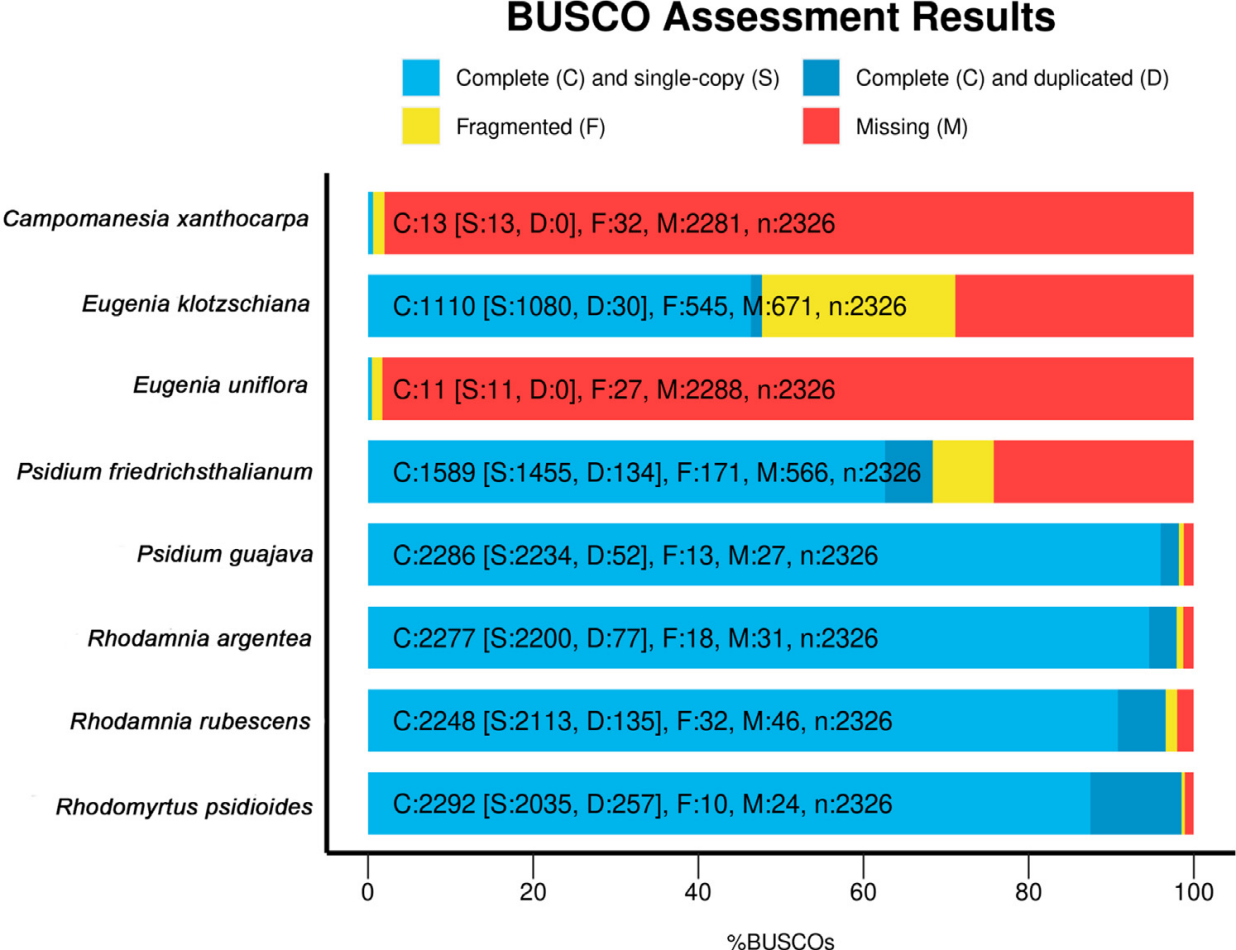
BUSCO assessment results for 8 Myrteae tribe genomes (Myrtaceae) using BUSCO version 5.3.2 with eudicots_odb10 database. The GenBank accession numbers for each species are: *Campomanesia xanthocarpa* (JAJKUD000000000.1); *Eugenia klotzschiana* (in this work and JANUWG000000000); *Eugenia uniflora* (RQIG00000000.1); *Psidium friedrichsthalianum* (JAGVVM000000000.1); *Psidium guajava* (JAEAMS000000000.1); *Rhodamnia argentea* (JAJJMR000000000.1); *Rhodamnia rubescens* (JAILYA000000000.1); *Rhodomyrtus psidioides* (JAILYB000000000.1).

The *E. klotzschiana* genome assembly has 75,180 contigs with a total size of 211,966,026 bp (Table 1) and 46.8% BUSCO completeness (Fig. 1). This assembly of *E. klotzschiana* shows greater completeness and less missing data than *E. uniflora*, making it a reference genomic resource for the genus *Eugenia*. However, there is a level of genome fragmentation that represents 23.4%, indicating the need for new sequencing approaches to obtain a more complete genome. Information about physical measurements of the genome size of *E. klotzschiana* is still lacking, but we calculated the predicted genome size based on the *k-mers* distribution for *E. klotzschiana* and found it to be of 282 Mb, meaning that the assembly provided on this work covers approximately 75.16% of the total expected genome size. The *E. klotzschiana* assembly has a genome depth of 12.98. This genome size is similar to other eight species of the Eugeniinae subtribe with an average of 258.74 Mbp estimated by flow cytometry [2]. The partial genome is available on NCBI JANUWG000000000 and the raw data is available on NCBI with the accession number SRR21159655. MISA identified 14,287 microsatellite regions, comprising 11,662 dinucleotides, 1,767 trinucleotides, 505 tetranucleotides, 193 pentanucleotides and 160 hexanucleotides. Additionally, using QDD, we isolated 9,087 sequences containing microsatellite regions, from which 327 sequences were filtered and used for primer design. We provide two sets of primers for SSR-Seq that were tested only *in silico*. The first set of primers (Ekl_SSR-Seq-1) is composed of 44 primers with minimum cross dimerization energy of -7ΔG (Table 2), of which 25 primer pairs (Ekl_SSR-Seq-2) show minimum cross-dimerization energy greater than -6ΔG and represent the second set of primers (Table 3). The available primers can be used in different Illumina sequencing platforms by adding specific platform tags, 5′-TCGTCGGCAGCGTCAGATGTGTATAAGACAG - 3′ for the Forward primers and for the Reverse primers, 5′- GTCTCGTGGGCTCGGAGATGTGTATAAGACAG - 3′. The use of SSR-Seq can accelerate genetic studies in natural populations because of rapid polymorphism identification. The validation of these sets of SSR-Seq primers on natural populations will allow us to investigate polymorphism both on size and on the nucleotide sequence of the repetitive units. Therefore, SSR-Seq is a resolving methodology for size homoplasy on microsatellite regions [3], due to misinterpretations of mutations that lead to identity by state and not identity by descent [4]. In addition, the use of high-throughput sequencing technology with SSR-Seq makes it possible to multiplex samples in a single sequencing run by using individual-specific barcoding, which can be applied to population genetics, increase the data, and reduce costs [5].

**Table 1:** Genome assembly statistics of the partial genome of *Eugenia klotzschiana*.

Parameter	Value
Number of contigs	75,180
Number of contigs ≥1000 bp	75,132
Total length	211,966,026 bp
Estimated genome size	282 Mb
Genome completeness	75.16%
Genome depth	12,98
Largest contig (bp)	113,288 bp
Shortest contigs (bp)	1000 bp
N50	3407
L50	19048
CG%	40.1

**Table 2:** First set of microsatellite primers (Ekl_SSR-Seq-1) with minimum cross dimerization > -7ΔG for *Eugenia klotzschiana* high-throughput genotype-by-sequencing.

Primer ID	Contig	SSR_Motif (number of repeats)	Primer Foward_5′-3′	Primer Reverse_5′-3′	Tm (°C)	PCR Frag length (bp)
Ekl_NGS1	33	ATCCG_7_	ACCTTAAACAAGTGAACCGT	TGAGCAAATTTACCCGATCA	49.80	156
Ekl_NGS2	785	AG_12_	TTGTACCCAAAGAAGAGCT	ACTCTCCTAAACGCCTTA	48.25	188
Ekl_NGS3	1218	AG_12_	GGAACGAAGCCTATCTCAAA	AATGGAGCAAATCGAAACAC	49.90	183
Ekl_NGS4	1502	AG_12_	ATAAGTGGGATACGAAAGC	TATGTAGTCAGCACACGAT	47.81	128
Ekl_NGS5	1656	AG_13_	CACCTATTGCCATTGGATCA	TGACCTCATGTTGCGATTTA	50.29	130
Ekl_NGS6	3138	AG_13_	AGTTTGAGAACCGAATCGA	GCTCTCTCAAACCATTCTGA	49.77	182
Ekl_NGS7	4085	AG_13_	TCACTCAAGTCAGCCTAAA	GGTCTTCAGAAGTTTGGGAT	49.45	181
Ekl_NGS8	6554	AG_13_	GAAAGGTCTTGATGTGCAT	AAGACAGAGGCAAGTTCATT	48.98	172
Ekl_NGS9	8176	AG_14_	AGGGCTTATATGGTAATCTCAC	ATGAGTCGTGTAAGCAACA	49.55	190
Ekl_NGS10	8525	AG_12_	TTTCTTTATCGGGTCACCTC	CTGATGCACCATTCCTCT	50.13	184
Ekl_NGS11	9082	AG_13_	TCTTCAATCGGCAGAAAGA	CCTTTCGCCCACCTAAATTA	49.97	184
Ekl_NGS12	9780	AACG_6_	CCTCCTAACAAGACTTGCAT	CCTTACCTTATGACGTCGTT	50.28	177
Ekl_NGS13	10665	AG_14_	AGAAGGGACTAGTGTAGCTT	CTGAATAGACAACCGAGGTT	50.30	171
Ekl_NGS14	12236	AG_13_	AAATCTGACAAGGGTGATCC	TGATGCTGACATGGATTTCT	50.00	138
Ekl_NGS15	13158	AG_12_	GAGCTCAAACCGAGATAGA	ATAGCTTTCCGACCAACA	49.17	188
Ekl_NGS16	13212	AG_12_	CGGGACATATAACAATCCGT	CCGTTGTTAATAGGGTCACA	50.34	136
Ekl_NGS17	13662	AG_13_	AATTCCAAAGTTCGTCCTCA	ATATGACCGATGTATGCTCC	49.75	194
Ekl_NGS18	15192	AG_13_	CAGTGATGTCTATCAGCTCC	CGCTAGTTTGAAGAGCAAA	49.72	169
Ekl_NGS19	15352	AG_13_	ACTACACGGTCAAGAAAGTC	AGCTTGGACCAGAAACTAT	49.45	168
Ekl_NGS20	18609	AG_14_	GAAGCTCCGTCTTACTCA	GCCAAACATCAAGTTCCA	48.95	195
Ekl_NGS21	20471	AG_13_	GGAGAAACAACCAGCTATGA	GGCTCTCAAAGTGAACCTTA	50.38	175
Ekl_NGS22	21084	AAGAG_6_	ACTTCTATGCGTTTGGATCA	GCTCTGGACTTCAATAGTGA	49.67	138
Ekl_NGS23	21751	AG_13_	TTACTCTGTCTTCTGGACCA	ATAAATGCAAGGACACCCAT	50.26	151
Ekl_NGS24	23261	AGGG_7_	AATTGACCAAGACGAAATGC	AGACTCTGACAAATTTCGCT	49.51	162
Ekl_NGS25	23736	AG_14_	GCACTTGGTCTCTACAAA	CCAGGAATTCAGCAGAGA	48.50	172
Ekl_NGS26	23844	AG_14_	CGTCCCTAACCAGAAGAATT	CGTCGAACACTTGAACAATT	49.69	198
Ekl_NGS27	28323	AG_12_	CAGTTCACCAGATTTAGCCT	CTCTCCCTGTTTCAATCAGT	50.27	137
Ekl_NGS28	28657	AG_12_	GGCCTCAAATGTCATCGATA	TGTTATGTCTGTGCCAATGA	50.14	188
Ekl_NGS29	29427	AG_12_	ATCGAGTTCTTTCAGTGCT	ATCAACGACTTCCAATGTGA	49.41	198
Ekl_NGS30	30205	AGGG_6_	GCAGGTAATCCAGTTTCAGA	AAATCTTCTGTTCACGTCGA	50.00	168
Ekl_NGS31	30515	AG_14_	CTGTGTTGCGTTAAACCA	GATTCGCGGTTATCGAAAT	48.60	192
Ekl_NGS32	32008	AG_13_	TCCGCATTTGGTACTACTT	AAGTCCTCTAGTCTGACC	48.51	196
Ekl_NGS33	33378	AG_12_	TCAAGAACTCATTCGAAGC	TTCACTTGTCTCTCCCATT	48.25	199
Ekl_NGS34	34669	AG_12_	AATCGCACTGATACCAAAGT	CAGTTTAGCTGTCTCATCGA	49.93	163
Ekl_NGS35	38622	ACAT_6_	ACTGAGCAATTGAAGTTGGA	CCCTGTTTCTTTCCTAGAC	49.00	165
Ekl_NGS36	54706	AG_12_	TCTGCTTGCGTATGATCATT	TATTACAGGGACAGCCGATA	50.25	191
Ekl_NGS37	56781	AG_13_	TTCCCTTTCGCACTCATAT	TTGGCTTCAAGAGAACTCT	48.90	143
Ekl_NGS38	60116	AG_14_	ATATTCTCTCTTTGCGGACC	CGAAACAAGTTGCATAGCTT	49.80	125
Ekl_NGS39	60721	AG_13_	TGAGACGTTGGAGTACTT	TTCCTAATGTTCCGCCATA	48.31	169
Ekl_NGS40	61462	AG_14_	ACTTGATGGACTTGCGATAA	CCTCAATCAGAATCCACGAT	49.87	147
Ekl_NGS41	62672	AG_12_	TCAGATCTCACAACATTGCA	AACCCATTGTCACTCTCTT	49.26	159
Ekl_NGS42	64383	AG_12_	GCCATCCTTGTAAACCCTA	CTCATGTCGAGTCTTCACA	49.93	196
Ekl_NGS43	67380	AG_12_	AAGAAGCGACTCCTTACAT	TCAGTTATCCCAAGTTCGAC	49.36	155
Ekl_NGS44	71322	AG_12_	AGTCAAAGCTGGGACAAA	CATCTCACAGACAACCAA	48.34	228
Maximum					50.38	228
Minimum					47.81	125
Delta					2.57	103

**Table 3:** Second set of microsatellite primers (Ekl_SSR-Seq-2) for for *Eugenia klotzschiana* high-throughput genotype-by-sequencing. This set of microsatellite primers was selected from Ekl_SSR-Seq-1 based on minimum cross dimerization > -6ΔG.

Primer ID	Contig	SSR_Motif (number of repeats)	Primer Foward_5′-3′	Primer Reverse_5′-3′	Tm (°C)	PCR Frag length (bp)
Ekl_NGS2	785	AG_12_	TTGTACCCAAAGAAGAGCT	ACTCTCCTAAACGCCTTA	48.26	188
Ekl_NGS3	1218	AG_12_	GGAACGAAGCCTATCTCAAA	AATGGAGCAAATCGAAACAC	49.91	183
Ekl_NGS5	1656	AG_13_	CACCTATTGCCATTGGATCA	TGACCTCATGTTGCGATTTA	50.30	130
Ekl_NGS6	3138	AG_13_	AGTTTGAGAACCGAATCGA	GCTCTCTCAAACCATTCTGA	49.77	182
Ekl_NGS7	4085	AG13	TCACTCAAGTCAGCCTAAA	GGTCTTCAGAAGTTTGGGAT	49.46	181
Ekl_NGS9	8176	AG_14_	AGGGCTTATATGGTAATCTCAC	ATGAGTCGTGTAAGCAACA	49.56	190
Ekl_NGS10	8525	AG_12_	TTTCTTTATCGGGTCACCTC	CTGATGCACCATTCCTCT	50.14	184
Ekl_NGS12	9780	AACG_6_	CCTCCTAACAAGACTTGCAT	CCTTACCTTATGACGTCGTT	50.28	177
Ekl_NGS13	10665	AG_14_	AGAAGGGACTAGTGTAGCTT	CTGAATAGACAACCGAGGTT	50.30	171
Ekl_NGS16	13212	AG_12_	CGGGACATATAACAATCCGT	CCGTTGTTAATAGGGTCACA	50.35	136
Ekl_NGS17	13662	AG_13_	AATTCCAAAGTTCGTCCTCA	ATATGACCGATGTATGCTCC	49.75	194
Ekl_NGS18	15192	AG_13_	CAGTGATGTCTATCAGCTCC	CGCTAGTTTGAAGAGCAAA	49.72	169
Ekl_NGS19	15352	AG_13_	ACTACACGGTCAAGAAAGTC	AGCTTGGACCAGAAACTAT	49.45	168
Ekl_NGS27	28323	AG_12_	CAGTTCACCAGATTTAGCCT	CTCTCCCTGTTTCAATCAGT	50.27	137
Ekl_NGS29	29427	AG_12_	ATCGAGTTCTTTCAGTGCT	ATCAACGACTTCCAATGTGA	49.42	198
Ekl_NGS30	30205	AGGG_6_	GCAGGTAATCCAGTTTCAGA	AAATCTTCTGTTCACGTCGA	50.01	168
Ekl_NGS31	30515	AG_14_	CTGTGTTGCGTTAAACCA	GATTCGCGGTTATCGAAAT	48.60	192
Ekl_NGS32	32008	AG_13_	TCCGCATTTGGTACTACTT	AAGTCCTCTAGTCTGACC	48.52	196
Ekl_NGS33	33378	AG_12_	TCAAGAACTCATTCGAAGC	TTCACTTGTCTCTCCCATT	48.26	199
Ekl_NGS34	34669	AG_12_	AATCGCACTGATACCAAAGT	CAGTTTAGCTGTCTCATCGA	49.94	163
Ekl_NGS35	38622	ACAT_6_	ACTGAGCAATTGAAGTTGGA	CCCTGTTTCTTTCCTAGAC	49.01	165
Ekl_NGS38	60116	AG_14_	ATATTCTCTCTTTGCGGACC	CGAAACAAGTTGCATAGCTT	49.80	125
Ekl_NGS39	60721	AG_13_	TGAGACGTTGGAGTACTT	TTCCTAATGTTCCGCCATA	48.31	169
Ekl_NGS41	62672	AG_12_	TCAGATCTCACAACATTGCA	AACCCATTGTCACTCTCTT	49.27	159
Ekl_NGS42	64383	AG_12_	GCCATCCTTGTAAACCCTA	CTCATGTCGAGTCTTCACA	49.94	196
Maximum					50.35	199
Minimum					48.26	125
Delta					2.09	74

## 2 Experimental Design, Materials and Methods

### 2.1 DNA Extraction and Sequencing

Leaves were collected from a single adult specimen of *E. klotzschiana* in a natural population from Senador Canedo - Goiás - Brazil (16°37′32,197" S, 49°4′22,696" W) with the voucher UFG0020120 deposited at Herbarium UFG. The samples were also registered on the National System for the Management of Genetic Heritage and Associated Traditional Knowledge (SisGen) under the authorization number A7D3EC4. Leaf tissues were dehydrated on silica gel and stored in a -80 °C freezer, following t otal DNA extraction with the CTAB protocol [6]. The DNA integrity was accessed using both agarose gel electrophoresis 1% and quantified using the Qubit 2.0 equipment (ThermoFisher). Genomic library was constructed with the SureSelectQXT kit (Agilent Technologies). Sequencing was performed on Illumina MiSeq platform, using MiSeq Reagent V3 kit (600 cycles), in the 2x300 bp paired-end mode.

### 2.2 Sequencing Quality Control and Genome Assembly

The base quality and sequence adapter presence on the raw data were analyzed using FastQC software (http://www.bioinformatics.babraham.ac.uk/projects/fastqc/). Read trimming was performed using Trimmomatic software [7] with parameters ILLUMINACLIP: Nextera-PE.fa:2:20:10, SLIDEWINDOW: 4:20, CROP: 263, HEADCROP: 21, and MINLEN:50, with a minimum mean Phred score of 20. The *de novo* assembly was performed using SPAdes assembler v3.15.4 [8]. The genome completeness was assessed using BUSCO v. 5.3.2 compared with eudicots database (eudicots_odb10) [9]. Additionally, seven other genomes from the Myrteae tribe (Myrtaceae) were obtained from the National Center for Biotechnology Information (NCBI) and analyzed using BUSCO, following the same pipeline. Comparative completeness was plotted considering all eight Myrteae species.

### 2.3 Gene Annotation, SSR Identification and Primer Multiplex Construction

Two approaches were used to characterize microsatellite regions. The first involves the use of MIcroSAtellite identification software [10,11] available https://webblast.ipk-gatersleben.de/misa/ for the identification and characterization of minimal repeats motif 10, 8, 6, 6 for dinucleotide, trinucleotides, tetranucleotide, pentanucleotide and hexanucleotide, respectively. Afterwards, the QDD software was used to select candidate regions for designing microsatellite primers in the context of SSR-Seq. The QDD software set the minimal repeats was the same applied with MISA software [12]. Primer development was done using Primer 3 software implemented in QDD software, defining the following parameters: (i) PCR product size between 120 and 200 bp; (ii) Primer size (minimum - optimal - maximum) of 18 bp - 20 bp - 23 bp; Melting temperature (minimum - optimal - maximum) of 48°C - 55°C - 62°C; (iv) Primer GC content (minimum - optimal - maximum) of 20% - 50% - 80%; (v) Maximum melting temperature difference 1 °C [12,^13^]. It was possible to design primers for 9,087 sequences, from which the primers that met the following criteria were removed: (i) trinucleotide SSR motif; (ii) SRR with repeats with 3 or more adenines in a row (AAA*); (iii) SSR motif with 100% AT content; (iv) SRR with distance of less than 20 bp from primers; (v) SRR in the context of transposable elements. After that, 327 SSRs with a primer pair survived the filter and were used to define a set of primers for multiplexing on FastPCR software [14]. From the single multiplex containing 99 primer pairs resulting from the FastPCR analysis, an *in silico* PCR was performed to evaluate the primer sets using openPrimeR [15] on Docker (https://hub.docker.com/r/mdoering88/openprimer/) . The assembled contigs were used as sequence templates and the designed primers were placed on a fasta file with forward and reverse sequences to be analyzed separately. Two sets of primers were tested, the first with minimum energy for cross-dimerization of -7ΔG (Ekl_SSR-Seq-1, Table 2) and the second including just primers with minimum energy for cross-dimerization of -6ΔG (Ekl_SSR-Seq-2, Table 3), which is a subset of the first set. For both sets the main settings for the evaluation and *in silico* PCR followed the program's default with some slight differences: optimal primer size was set to 18–22 bp, allowed mismatches between the primer sequence and the template were 5 bp and mismatches were forbidden on the last 6 bp of the primer's 3′ end. After the evaluation, primers bound to other regions rather than the target ones, primers set with only one primer orientation binding to the target region, and primers that did not fulfill the desired physicochemical properties were filtered and discarded. The remaining primers were chosen to build two multiplex sets presented here on Tables 2 and 3. For genotype-by-sequence on Illumina platforms, it is necessary to add specific tags to the primers, 5′-TCGTCGGCAGCGTCAGATGTGTATAAGACAG - 3′ in the Forward primers and in the Reverse primers 5′- GTCTCGTGGGCTCGGAGATGTGTATAAGACAG - 3′.

## Ethics Statements

N/A.

## CRediT authorship contribution statement

**Leonardo C.J. Corvalán:** Writing – original draft, Methodology, Formal analysis, Investigation, Data curation, Visualization. **Larissa R. Carvalho:** Writing – original draft, Methodology, Formal analysis, Investigation, Data curation, Visualization. **Amanda A. Melo-Ximenes:** Writing – original draft, Formal analysis, Data curation. **Cíntia P. Targueta:** Methodology, Resources, Writing – review & editing. **Ramilla S. Braga-Ferreira:** Methodology, Resources, Writing – review & editing. **Rhewter Nunes:** Conceptualization, Writing – review & editing, Supervision, Project administration. **Mariana P.C. Telles:** Conceptualization, Writing – review & editing, Supervision, Funding acquisition.

## Declaration of Competing Interest

The authors declare that they have no known competing financial interests or personal relationships that could have appeared to influence the work reported in this paper.

## Data Availability

Data on the draft genome assembly of Eugenia klotzschiana (Myrtaceae) (Original data) (NCBI and in the paper).

## References

[1] Vieira R.F., Camillo J., Coradin L. (2016).

[2] Costa I.R., Dornelas M.C., Forni-Martins E.R. (2008). Nuclear genome size variation in fleshy-fruited Neotropical Myrtaceae. Plant Syst. Evol..

[3] Šarhanová P., Pfanzelt S., Brandt R., Himmelbach A., Blattner F.R. (2018). SSR-seq: Genotyping of microsatellites using next-generation sequencing reveals higher level of polymorphism as compared to traditional fragment size scoring. Ecol. Evol..

[4] Estoup A., Jarne P., Cornuet J.M. (2002). Homoplasy and mutation model at microsatellite loci and their consequences for population genetics analysis. Mol. Ecol..

[5] Baggett J.P., Tillett R.L., Cooper E.A., Yerka M.K. (2021). De novo identification and targeted sequencing of SSRs efficiently fingerprints Sorghum bicolor sub-population identity. PLoS One.

[6] Doyle J.J., Doyle J.L. (1987). A rapid DNA isolation method for small quantities of fresh tissues. Phytochem. Bull..

[7] Bolger A.M., Lohse M., Usadel B. (2014). Trimmomatic: a flexible trimmer for Illumina sequence data. Bioinformatics.

[8] Bankevich A., Nurk S., Antipov D., Gurevich A.A., Dvorkin M., Kulikov A.S., Lesin V.M., Nikolenko S.I., Pham S., Prjibelski A.D., Pyshkin A.V., Sirotkin A.V., Vyahhi N., Tesler G., Alekseyev M.A, Pevzner P.A. (2012). SPAdes: a new genome assembly algorithm and its applications to single-cell sequencing. J. Comput. Biol..

[9] Manni M., Berkeley M.R., Seppey M., Simão F.A., Zdobnov E.M. (2021). BUSCO update: novel and streamlined workflows along with broader and deeper phylogenetic coverage for scoring of eukaryotic, prokaryotic, and viral genomes. Mol. Biol. Evol..

[10] Beier S., Thiel T., Münch T., Scholz U., Mascher M. (2017). MISA-web: a web server for microsatellite prediction. Bioinformatics.

[11] Thiel T., Michalek W., Varshney R., Graner A. (2003). Exploiting EST databases for the development and characterization of gene-derived SSR-markers in barley (Hordeum vulgare L. Theor. Appl. Genet..

[12] Meglécz E., Pech N., Gilles A., Dubut V., Hingamp P., Trilles A., Grenier R., Martin J.F. (2014). QDD version 3.1: a user friendly computer program for microsatellite selection and primer design revisited: experimental validation of variables determining genotyping success rate. Mol. Ecol. Resources.

[13] Untergasser A., Cutcutache I., Koressaar T., Ye J., Faircloth B.C., Remm M., Rozen S.G. (2012). Primer3-new capabilities and interfaces. Nucleic Acids Res..

[14] Kalendar R., Khassenov B., Ramankulov Y., Samuilova O., Ivanov K.I. (2017). FastPCR: An in silico tool for fast primer and probe design and advanced sequence analysis. Genomics.

[15] Kreer C., Doring M., Lehen N., Ercanoglu M.S., Gieselmann L., Luca D., Jain K., Schommers P., Pfeifer N., Klein F. (2020). openPrimeR for multiplex amplification of highly diverse templates. J. Immunol. Methods.

